# Relationship Between Periodontitis and Nitric Oxide in Patients Undergoing Maintenance Hemodialysis

**DOI:** 10.1111/hdi.13264

**Published:** 2025-05-26

**Authors:** Gabriela Keiko Izumi, Caroline Vidal Paseto, Rafael Fiorese Costa, Gabrielle Delfrate, Aline Borsato Hauser, Daniel Fernandes, Maria Ângela Naval Machado, Reila Tainá Mendes

**Affiliations:** ^1^ Department of Stomatology Postgraduate Programme in Dentistry, Federal University of Paraná Curitiba Paraná Brazil; ^2^ Foundation Pró‐Renal Curitiba Paraná Brazil; ^3^ Department of Pharmacology Federal University of Santa Catarina Florianópolis Santa Catarina Brazil; ^4^ Department of Clinical Analysis Pharmacy Course, Federal University of Paraná Curitiba Paraná Brazil

**Keywords:** chronic renal insufficiency, inflammation mediators, nitric oxide, periodontal diseases, renal dialysis

## Abstract

**Background:**

The inflammatory processes associated with periodontitis have been implicated in the development and progression of systemic diseases, including chronic kidney disease (CKD). Notably, individuals with CKD frequently exhibit shared risk factors with those affected by periodontitis, such as hypertension, smoking, and diabetes mellitus. Nitric oxide (NO), a free radical with diverse physiological and pathological roles, exerts both anti‐inflammatory (e.g., vasodilation, modulation of platelet function) and pro‐inflammatory effects, the latter of which have been observed in patients with CKD.

**Aims:**

This study aimed to investigate the relationship between nitric oxide levels in saliva and plasma and various periodontal parameters in patients with chronic kidney disease (CKD), in comparison to a control group without CKD.

**Methods:**

This study enrolled 90 participants seeking dental treatment. The participants were divided into two groups: a CKD group (*n* = 40) consisting of patients undergoing dialysis or hemodialysis treatment at the Pró‐Renal Foundation Dental Clinic, and a control group (*n* = 50) comprising individuals without CKD who were receiving treatment at the UFPR Dental Clinic. Two calibrated examiners conducted comprehensive periodontal examinations for all participants. Additionally, saliva samples were collected from each participant to assess pH, flow rate, and nitric oxide levels. Venous blood samples were also obtained to quantify plasma nitric oxide concentrations.

**Results:**

Patients in the CKD group exhibited significantly poorer periodontal health compared to the control group (*p* < 0.05), characterized by a larger Periodontal Inflamed Surface Area, greater probing depth, increased clinical attachment level, and a higher visual plaque index (*p* < 0.05). Furthermore, the CKD group presented with significantly reduced salivary flow (*p* < 0.05). Notably, this group also showed elevated levels of both nitrate and nitrite in saliva and serum (*p* < 0.05) compared to the control group. Correlation analyzes revealed significant positive associations between nitrate and nitrite levels (in both plasma and saliva) and several periodontal variables: probing depth (*r* = 0.38 and 0.41, respectively), clinical attachment level (*r* = 0.40 and 0.38, respectively), and Periodontal Inflamed Surface Area (*r* = 0.27 and 0.48, respectively). Conversely, nitrate and nitrite levels in both fluids showed significant negative correlations with glomerular filtration rate (GFR) (*r* = −0.55 and −0.40, respectively). The visual plaque index demonstrated a significant positive correlation only with salivary nitrate and nitrite levels (*r* = 0.23), highlighting the potential influence of oral biofilm on nitric oxide metabolism. Interestingly, the comparable levels of nitrate and nitrite observed in saliva and plasma suggest that saliva may be a suitable non‐invasive biofluid for biomarker analysis in this context.

**Conclusion:**

This study revealed a positive correlation between the visual plaque index and salivary nitric oxide levels in the studied population. However, further research involving specific analysis of the oral biofilm composition and its dynamic role within the nitric oxide entero‐salivary cycle in these patients is warranted to elucidate this relationship fully.

## 1 Introduction

A bi‐directional relationship between chronic kidney disease (CKD) and periodontitis has been well documented [1]. CKD is strongly associated with increased morbidity and mortality from non‐communicable diseases [2]. Globally, an estimated 850 million individuals are affected by CKD, which resulted in 1.2 million deaths in 2017. Several risk factors, including impaired fasting plasma glucose and hypertension, have been implicated in the development and progression of CKD [2]. Furthermore, the systemic inflammation characteristic of periodontal disease has been proposed as a plausible biological mechanism underlying its association with other systemic conditions [3].

Periodontal disease is an infectious and inflammatory disease associated with a dysbiotic biofilm that progressively affects the surrounding tissues of the teeth [4]. Without treatment, this results in alveolar bone resorption, potentially causing tooth loss and subsequent masticatory dysfunction, as well as impairments in speech, esthetics, and overall quality of life. Furthermore, periodontal disease places a significant financial burden on healthcare systems [5, 6]. The global estimated prevalence of severe periodontal disease was 18% in 2019 [7].

The exaggerated inflammatory response in periodontal disease leads to an increased release of chemokines, cytokines, and proteinases. Consequently, the gingiva exhibits elevated levels of interleukins, including Interleukin‐1β, Interleukin‐6, and Interleukin‐8 [8]. Beyond local inflammation, periodontal disease is recognized as a significant infectious focus that contributes to increased systemic inflammation and morbidity of affected patients [9, 10]. Furthermore, it is classified within the group of chronic Noncommunicable Diseases. Periodontal disease also shares common risk factors with major Noncommunicable Diseases, including diabetes, heart disease, chronic respiratory disease, and CKD [5, 11, 12]. The association between periodontal disease and chronic Noncommunicable Diseases may be partly explained by endothelial dysfunction, a condition characterized by reduced vasodilator capacity due to diminished nitric oxide bioavailability [12, 13, 14, 15]. Conversely, inflammatory states can potentially compensate for the lack of nitric oxide bioavailability through the overexpression of the inducible form of nitric oxide synthase. When induced, this enzyme produces large amounts of nitric oxide, which can exert cytotoxic effects [16].

Periodontal disease has previously been identified as a significant risk factor for patients with kidney failure. Both conditions share a common hyper‐inflammatory profile, characterized by elevated concentrations of pro‐inflammatory cytokines (such as C‐reactive protein and Interleukins −2 and −6) and endothelial dysfunction [15, 17, 18, 19]. It is hypothesized that the microbiota associated with periodontal inflammation may disrupt the host immune response, thereby facilitating the development of pathology or complications at systemic sites [20]. The microbial imbalance could contribute to the chronic systemic inflammatory burden, potentially exacerbating the condition of patients undergoing maintenance hemodialysis [21].

Chronic renal failure, or CKD, is a condition characterized by the progressive and often irreversible loss of normal kidney function, accompanied by structural hypertrophy of the remaining nephrons; it can have tubular, glomerular, or endocrine origins [22]. CKD is frequently associated with other systemic changes, such as hypertension and diabetes, and a significant proportion of patients with the disease exhibit periodontal inflammation [23]. Recent evidence suggests that periodontitis is associated with a worsening of CKD status through increased oxidative stress and cytokine release; conversely, the inflammatory profile characteristic of CKD is also linked to poor oral health [24]. Nevertheless, further research is needed to fully understand the intricate relationship between these two diseases.

Numerous observational studies have demonstrated associations between periodontitis and various systemic diseases, including cardiovascular diseases [25, 26, 27]; diabetes mellitus [28, 29, 30]; Alzheimer's disease [31]; CKD [17, 23, 32]; and alterations in arterial and intracranial pressure [33]. The imbalance of the local microbiota and consequent dysbiosis have already been demonstrated in several studies [10] to explain the local consequences of periodontitis. However, the contribution of periodontitis to the pathogenesis of other systemic diseases warrants further investigation. To our knowledge, the specific association between periodontal status and CKD parameters remains to be fully elucidated. Therefore, this study aims to investigate the relationship between nitric oxide levels (in plasma and saliva) and clinical periodontal parameters in patients with and without CKD.

## 2 Materials and Methods

### 2.1 Ethical Considerations and Study Design

This study was approved by the Ethics Committee in Research of the Federal University of Paraná (CEP/SD 4.912.265).

It adhered to the principles outlined in the Declaration of Helsinki and the STROBE (Strengthening The Reporting of Observational Studies in Epidemiology) guidelines for cross‐sectional case‐control studies. All participants provided their written informed consent.

### 2.2 Study Population

The study population comprised two groups: a case group (*n* = 40) consisting of patients with CKD undergoing peritoneal dialysis or hemodialysis, who were receiving care at the dental clinic of the Pró‐Renal Foundation (Curitiba, PR); and a control group (*n* = 50) of patients without CKD, who were receiving care at the dental clinic of the Federal University of Paraná (Curitiba, PR).

The sample calculation was based on salivary nitric oxide values reported in previous literature [34]. Considering mean ± SD values of 44.45 ± 26.89 μmol/L for the control group and 584.31 ± 510.7 μmol/L for the CKD group, a power of 95% and a type I error rate of 5%, the GPower software [35] determined a required sample size of 21 patients per group. To account for a potential 12% non‐compliance rate in both groups, the sample size was adjusted to 80 patients. The study and control groups were matched for age (*p* < 0.05).

### 2.3 Inclusion Criteria

Participants in both groups were individuals aged 18 or older, of either gender, with at least six teeth, who sought care at either the Dental Clinic of the Pró‐Renal Foundation (Curitiba—Paraná) for the case group or the Federal University of Paraná for the control group.

### 2.4 Exclusion Criteria

Patients were excluded if they were current smokers or former smokers for less than 5 years, pregnant women, had received periodontal treatment within the past 3 months, or had cognitive impairments hindering cooperation.

### 2.5 Renal Function of the Control Group

Plasma creatinine levels were analyzed at LEAC (Laboratório Escola de Análises Clínicas at UFPR) to assess renal health. Normal creatinine values were defined as < 1.3 mg/dL for men and < 1.0 mg/dL for women.

### 2.6 Data Collection

Sociodemographic data, medical history, and oral hygiene habits were collected from both groups through anamnesis.

### 2.7 Physical Examination of Periodontal Disease

Two trained examiners (GKI and CVP) conducted periodontal examinations on a separate group of eight patients, who were not included in the final study sample, to assess intra‐ and inter‐examiner reproducibility for clinical attachment level and periodontal probing depth measurements. All teeth were examined. Each examiner took duplicate measurements with a one‐week interval between examinations. The agreement between measurements was analyzed using the Bland–Altman graphic method. The majority of points fell within the limits of agreement, with a bias of 0.11 ± 0.62 mm. This bias was within acceptable reproducibility tolerance of ±1.0 mm.

Six sites per tooth were probed using a North Carolina millimeter periodontal probe. Probing depth, clinical attachment level, and bleeding on probing were the assessed parameters. Periodontal conditions were classified according to Caton et al. [4]. The Periodontal Inflamed Surface Area index was calculated in mm [2] to quantify the area of inflamed periodontal tissue [36]. The calculation utilized periodontal examination data transferred to a Microsoft Excel spreadsheet, as described by Nesse et al. [36].

### 2.8 Saliva Collection

Saliva samples were collected at two different time points. Unstimulated saliva was collected by allowing it to accumulate for 3 min before expectoration into a 50 mL sterile flask. Following a one‐minute interval, stimulated saliva was collected by having patients expectorate into another 50 mL flask after using a standardized rubber tourniquet (1.0 × 0.5 cm) tied with dental floss. Both collections occurred on the same morning. A 200 μL aliquot of the stimulated saliva sample was then transferred using a 1 mL micropipette to a 1.5 mL eppendorf tube and stored at −80°C for subsequent nitrate and nitrite analysis. Unstimulated saliva was used to analyze salivary flow rate and pH.

### 2.9 Quantification of Nitrite and Nitrate

Salivary and plasma nitrite and nitrate levels were quantified using the methodology described by Granger et al. [37] The Granger method, based on the Griess reaction, has been a standard for measuring nitrate and nitrite (the major end products of nitric oxide oxidation) in biological samples since 1996 [38]. Given the very short half‐life of nitric oxide in biological systems, quantifying its stable end products (nitrate and nitrite) is a well‐established and reliable approach for estimating nitric oxide production [39]. We chose this method due to its simplicity, cost‐effectiveness, and our prior experience confirming its reliability and reproducibility under similar experimental conditions. Briefly, nitrate was converted to nitrite by incubating samples at 37°C for 2 h with nitrate reductase expressed in *Escherichia coli* grown anaerobically. Following incubation, samples were centrifuged to remove the bacteria, and 100 μL of the supernatant was mixed (1:1) with Griess reagent in a 96‐well plate. Absorbance was read at 540 nm using a plate reader (ELISA). Nitrite and nitrate standard curves (0–150 μM) were run concurrently. Finally, linear regression was used to express the values as μM of NOx (nitrite and nitrate).

Saliva and serum were compared due to low cost, non‐invasive nature, simplicity, and painless collection of saliva. These characteristics make saliva a promising fluid for biomarker detection and clinical applications. Moreover, while some biomarkers are found in both blood and saliva, salivary levels are often higher [40].

### 2.10 Statistical Analysis

Data are presented as mean ± SD or median ± SE, and as total numbers or percentages, as specified in table legends.

The normality of quantitative variables was assessed using the Kolmogorov–Smirnov test. Parametric data were analyzed with Student's *t*‐test, whereas non‐parametric data were analyzed with the Mann–Whitney test.

Qualitative variables were analyzed using the chi‐squared test.

Spearman's correlation coefficient was calculated to assess the relationships between nitrate and nitrite levels (in both saliva and plasma), clinical periodontal parameters, and glomerular filtration rate.

A *p* value of less than 0.05 was considered statistically significant. All analyses were performed using GraphPad Prism version 8 software.

## 3 Results

### 3.1 Characterization of the Sample and Sociodemographic Variables

A total of 90 patients were included in this study: 40 with CKD (Stage 5) and 50 in the control group (without CKD). Sociodemographic data and general health conditions for both groups are presented in Table 1. To confirm renal health of the control group, their plasma creatinine levels were checked (data not shown).

**TABLE 1:** Sociodemographic variables of patients with or without chronic kidney disease.

Variable	Control (*n* = 50)	CKD (*n* = 40)	*p*
Age (mean ± SD)^a^	43.38 ± 15.56	47.88 ± 14.45	0.15
Gender *n* (M/F)^b^	23/27	31/9	0.002
Color (White/brown or black)^b^	40/10	25/15	0.065
Diabetes *n* (%)	1 (2%)	13 (32%)	< 0.0001
Hypertension *n* (%)	6 (12%)	32 (80%)	< 0.0001

Abbreviations: F, female; M, male; *n*, number of individuals; SD, standard deviation.

^a^ Normal distribution of data using theKolmogorov‐Smirnov test; data analyzed using the *t*‐test.

^b^ Data analyzed by chi‐square test.

### 3.2 Periodontal Conditions

Periodontal conditions were assessed using two methods: the current classification of periodontal diseases [4, 41]; and the Periodontal Inflamed Surface Area index, which quantifies the area of inflammation of periodontal tissue [36]. The results of these assessments are presented in Table 2.

**TABLE 2:** Periodontal conditions and oral hygiene habits of patients with and without chronic kidney disease.

Variable	Control (*n* = 50)	CKD (*n* = 40)	*p*
PISA median ± SE (25th–75th percentil) (mm^2^)^a^	7.97 ± 13.81 (0.00–34.30)	90.37 ± 34.49 (25.97–219.40)	0.0003
Periodontal conditions (*n*/%)^b^	Health: 37/74% Periodontitis stages I or II: 7/14% Periodontitis stages III or IV: 6/12%	Health: 1/3% Periodontitis stages I or II: 15/37% Periodontitis stages III or IV: 24/60%	< 0.0001
PD median ± SE (25th–75th percentil)^c^ (mm)	1.57 ± 0.09 (1.34–1.80)	3.34 ± 0.18 (1.72–3.90)	< 0.001
CAL median ± SE (25th–75th percentil)^c^ (mm)	1.69 ± 0.12 (1.44–2.22)	3.34 ± 0.16 (2.36–3.96)	< 0.001
VPI median ± SE (25th–75th percentil) (%)^c^	10.50 ± 4.09 (0.00–16.00)	27.00 ± 5.45 (8.00–60.00)	0.0002
Number of teeth in mouth median ± SE (25th–75th percentil)^c^	27.00 ± 0.84 (23.00–28.00)	20.00 ± 1.16 (12.00–26.00)	< 0.0001
Brushing (*n*)^b^ (up to 2×/3× or more)	16/34	17/23	0.304
Flossing (*n*)^b^ (No/Yes)	24/36	30/10	0.009

Abbreviations: CAL, clinical attachment loss; *n*, number of individuals; PD, probing depth; PISA, periodontal inflamed surface area; SE, standard error; VPI: visible plaque index.

^a^ Data analyzed using the *t*‐test.

^b^ Data analyzed using the chi‐squared test.

^c^ Data analyzed using Mann–Whitney test.

### 3.3 Saliva

Table 3 presents the results obtained from the salivary analysis, specifically pH and flow rate.

**TABLE 3:** Salivary flow and pH of patients with and without chronic kidney disease.

Variable	Control (*n* = 50)	CKD (*n* = 40)	*p*
Unstimulated salivary flow median ± SE (25th–75th percentil) (g/min)	0.60 ± 0.06 (0.37–0.90)	0.60 ± 0.060 (0.30–0.80)	0.400
Stimulated salivary flow median ± SE (25th–75th percentil) (g/min)	1.25 ± 0.09 (0.90–1.72)	0.90 ± 0.09 (0.50–1.30)	0.006
pH median ± SE (25th–75th percentil)	7.50 ± 0.06 (7.17–7.82)	7.60 ± 0.37 (7.00–8.50)	0.480

*Note*: Data expressed as median ± SE (25th–75th percentil). Data analyzed by Mann–Whitney test.

Abbreviation: *n*, number of individuals.

### 3.4 Quantification of Nitrate and Nitrite (NOx)

Table 4 presents the salivary and plasma nitrate and nitrite values. Data on the consumption of leafy greens and beetroot, which can influence these levels, were also recorded.

**TABLE 4:** Quantification of nitrite and nitrate (NOx) in saliva and serum of patients with and without chronic kidney disease.

Variable	Control (*n* = 47)	CKD (*n* = 34)	*p*
NOx salivary^a^ (μmol/L)	196.5 ± 138.0 (87.83–308.5)	573.7 ± 399.5 (231.0–838.3)	< 0.0001
NOx serum^a^ (μmol/L)	11.03 ± 11.10 (4.28–14.98)	41.58 ± 25.40 (21.79–51.09)	< 0.0001
Leafy greens (Yes/No) (*n*)^b^	29/18	17/17	0.294

Abbreviation: *n*, number of individuals.

^a^ Data expressed as mean ± SD (25th–75th percentile) and analyzed by *t*‐test.

^b^ Data analyzed by chi‐square test.

### 3.5 Correlation Coefficients for Clinical Parameters and Nitrate and Nitrite in Saliva and Plasma Samples

To investigate the association between nitric oxide and clinical parameters in periodontal disease, a correlation analysis was performed, and the results are presented in Table 5.

**TABLE 5:** Correlation coefficients for clinical parameters and nitric oxide in saliva and plasma samples using Spearman's rank correlation test.

	PISA	PD	CAL	VPI	GFR	NOx saliva	NOx plasma
NOx (plasma)	*r* = 0.274 *p* = 0.01	*r* = 0.384 *p* = 0.0009	*r* = 0.407 *p* = 0.0004	*r* = 0.096 *p* = 0.42	*r* = −0.553 *p* < 0.0001	*r* = 0.376 *p* = 0.002	—
NOx (saliva)	*r* = 0.481 *p* < 0.0001	*r* = 0.418 *p* < 0.0001	*r* = 0.382 *p* < 0.0001	*r* = 0.232 *p* = 0.03	*r* = −0.408 *p* = 0.0003	—	*r* = 0.376 *p* = 0.002

Abbreviations: CAL, clinical attachment level; GFR, glomerular filtration rate; NOx, nitrate and nitrite; PD, probing depth; PISA, periodontal inflamed surface area; VPI, visible plaque index.

## 4 Discussion

This study investigated the correlation between nitric oxide levels (salivary and plasmatic) and clinical periodontal parameters in patients with and without CKD. To our knowledge, this is the first study to associate nitric oxide levels in both saliva and plasma in individuals with CKD and periodontitis. We found higher nitrate and nitrite levels in the saliva and plasma of patients with CKD, which aligns with previous findings reporting elevated levels in saliva [34] and plasma [42] of patients with CKD. Furthermore, nitrate and nitrite levels showed a significant correlation with periodontal parameters (Periodontal Inflamed Surface Area, probing depth, and clinical attachment level) and with GFR, indicating a strong relationship between periodontal disease and CKD.

Salivary and plasmatic levels were analyzed to assess the feasibility of using saliva as a less invasive and more cost‐effective alternative to plasma analysis. Like other biological fluids, saliva contains a rich array of biomolecules, including DNA, mRNA, proteins, metabolites, and microbiota. These properties, coupled with its ease of collection, make saliva a valuable fluid for research [43]. Interestingly, the visible plaque index showed a significant positive correlation only with salivary nitrate and nitrite, highlighting the role of oral biofilm metabolism in the entero‐salivary nitrate cycle. Salivary and plasma nitrate and nitrite levels exhibited similar correlations with the other variables examined.

Despite similar self‐reported oral healthcare habits (brushing and flossing frequency) between the groups, our results revealed significantly worse oral conditions in CKD patients compared to the control group. This was evidenced by a higher Periodontal Inflamed Surface Area index (*p* < 0.05), greater visible plaque index (*p* < 0.05), fewer teeth in the mouth (*p* < 0.05), and increased mean probing depth and clinical attachment level (*p* < 0.05). These findings align with existing literature demonstrating poorer periodontal health, greater attachment loss, and alveolar bone loss in CKD patients. Notably, this inflammatory state is linked to a faster decline in kidney function [17, 44, 45, 46, 47, 48, 49]. Only one patient in the CKD group had a healthy periodontium, compared to 26% of patients in the control group who had periodontitis (97% in the CKD group, *p* < 0.05). The compromised oral health observed in the CKD group underscores the necessity of multidisciplinary treatment for individuals with chronic diseases and emphasizes periodontal treatment as a crucial component in the management of CKD patients.

Salivary alternations can also contribute to the worse oral conditions observed in CKD patients. Xerostomia, halitosis, and a burning sensation on the tongue are frequently reported during CKD [48]. In this study, CKD patients exhibited a significant reduction in stimulated salivary flow (*p* < 0.05), potentially linked to a low water intake, medication use, salivary gland alterations, and glomerular disease resulting from uremia [49, 50, 51]. Another common salivary change in CKD patients is a more alkaline pH, attributed to fluctuations in urea levels, medication use (antidepressants, diuretics, gastric drugs), and a diet rich in green foods [52]. Alkaline salivary pH is known to promote periodontal disease progression [53]. However, in our study, salivary pH did not differ significantly between the two groups (*p* > 0.05).

Given that both periodontitis and CKD are systemic inflammatory conditions, nitric oxide likely plays a significant role in their pathophysiology. Existing literature indicates altered nitrate and nitrite levels in patients with periodontitis [54, 55, 56, 57, 58] and those with CKD [34, 42]. An observational study of 45 CKD patients reported a decrease in nitrate excretion with declining kidney function [42]. In periodontitis, reactive oxygen species from neutrophil hyperactivity can overwhelm antioxidant defenses, leading to periodontal tissue destruction and increased local and systemic oxidative stress via endothelial dysfunction [59]. Nitrates may also influence the oral microbiota balance, promoting symbiosis [60], and nitrite‐producing bacteria have been shown to increase post‐periodontal treatment [61]. Consistent with this, our findings revealed higher salivary and plasma nitrate and nitrite levels (*p* < 0.05) in the CKD group, with these levels showing a negative correlation with GFR. It is important to note that elevated nitric oxide levels do not necessarily equate to higher nitrate and nitrite bioavailability, as inflammation can induce overexpression of inducible nitric oxide synthase, leading to the production of large amounts of nitric oxide with cytotoxic effects [16].

It is noteworthy that inorganic nitrate is abundant in vegetables, particularly leafy greens and beetroot. Upon ingestion, nitrate is rapidly absorbed in the intestines and enters the circulation. Although most nitrate is excreted via urine, approximately 25% is secreted through the salivary glands [62]. Consequently, a patient's diet can impact nitrate levels. In this study, self‐reported consumption of nitrate‐rich foods did not differ significantly between individuals with and without CKD (*p* > 0.05), allowing us to exclude diet as a confounding factor in our results.

As previously mentioned, whereas the majority of ingested nitrate is excreted in the urine, approximately 25% is eliminated through the salivary glands [62]. In saliva, commensal bacteria reduce nitrate to nitrite [63]. Sequentially, upon swallowing, a portion of this nitrite is further reduced to nitric oxide in the acidic environment of the stomach, allowing a significant amount of nitrite to enter the circulation [64]. Circulating nitrite can then be reduced to nitric oxide by vascular nitrite reductases [65]. This bioactivation pathway of nitrate has several beneficial biological effects, including blood pressure reduction and anti‐inflammatory action [64, 66]. Thus, this non‐enzymatic route could potentially help restore nitric oxide levels during endothelial dysfunction [67, 68]. Conversely, it is crucial to consider that high levels of nitric oxide, typically produced by inducible nitric oxide synthase during inflammation, can also be associated with detrimental conditions such as kidney failure [16, 42, 69]. In our study, we observed a negative correlation between GFR and both salivary and plasma nitrate and nitrite levels, indicating that lower GFR values were associated with higher nitrate and nitrite levels (*p* < 0.05).

Cross‐sectional studies have demonstrated increased salivary and plasma levels of nitric oxide in patients with periodontitis [54, 55, 56]. In a preclinical study investigating the correlation between nitric oxide, endothelial function, and periodontitis, mice with experimental periodontitis were administered water containing potassium nitrate. The results showed that animals receiving potassium nitrate exhibited reduced gingival inflammation, decreased gingival expression of Interleukin‐1 beta, and increased expression of Interleukin‐10. However, no significant differences were observed in alveolar bone loss or oral biofilm composition between the groups that received or did not receive potassium nitrate [15]. These findings contrast with changes observed in the biofilm of patients who consumed or did not consume a high‐nitrate diet [70]. Notably, our findings revealed a positive correlation between the visible plaque index and salivary (but not plasmatic) nitric oxide levels. This observation underscores the significant role of oral biofilm in nitric oxide metabolism and highlights the necessity for further research to elucidate the precise mechanisms of oral biofilm involvement in nitric oxide metabolism during periodontitis.

One limitation of our study is the imbalance in sex distribution between the groups, with more men in the CKD group [31] compared to the control group [23], which could potentially have influenced our findings. However, we conducted additional analyzes excluding female participants from the control group, and these analyzes yielded no significant differences (data not shown). Therefore, we opted to present the data from the entire sample. Another potential limitation is the recruitment of participants from dental clinics. This convenience sampling approach means that our study population may not be fully representative of the broader population seeking or not seeking oral care.

## 5 Conclusion

In conclusion, our findings underscore the necessity of interdisciplinary treatment for patients with chronic diseases like CKD, given their poorer oral health characterized by more advanced periodontitis and a reduced number of teeth in the mouth. Effective management of periodontitis in this population may represent a crucial step in mitigating systemic inflammation. Achieving a symbiotic biofilm could be vital for restoring balance in the nitrite‐nitrate‐nitric oxide cascade, potentially serving as a preventive strategy for prevalent chronic inflammatory diseases, including CKD and periodontitis. The similar levels of nitric oxide observed in saliva and plasma suggest saliva as a promising non‐invasive fluid for biomarker analysis. Finally, the positive correlation between the visible plaque index and salivary nitric oxide levels highlights the need for specific analyzes of the oral biofilm of these patients and its role in the nitric oxide entero‐salivary cycle.

## Conflicts of Interest

The authors declare no conflicts of interest.

## Data Availability

The data that support the findings of this study are available on request from the corresponding author. The data are not publicly available due to privacy or ethical restrictions.
